# Probiotics Cancer Interaction, Prevention, and Therapy

**DOI:** 10.1002/iid3.70435

**Published:** 2026-05-14

**Authors:** Mostafa Saebi, Amir Hossein Barjasteh, Mahmoud Mahmoudi, Ramiar Kamal Kheder, Setayesh Ramazanpour, Negar Ebadpour, Afsane Fadaee, Seyed‐Alireza Esmaeili

**Affiliations:** ^1^ Immunology Research Center Mashhad University of Medical Sciences Mashhad Iran; ^2^ Student Research Committee Mashhad University of Medical Sciences Mashhad Iran; ^3^ Immunology Department, Faculty of Medicine Mashhad University of Medical Sciences Mashhad Iran; ^4^ Medical Laboratory Science Department, College of Science University of Raparin Rania Sulaymaniyah Iraq; ^5^ Department of Medical Analysis, Faculty of Applied Science Tishk International University Erbil Iraq

**Keywords:** cancer prevention, cancer therapy, chemotherapy, gut microbiota, probiotics

## Abstract

Cancer is a significant global health concern and ranks as the second most common cause of death on a worldwide scale. A combination of hereditary and lifestyle variables, such as diet, smoking, alcohol consumption, physical exercise, and the gut microbiota, impacts cancer development. Dysbiosis, an imbalance in gut microorganisms, can promote cancer. Conversely, probiotics have the potential to enhance gut health and restore immunological equilibrium. Prior research has demonstrated that probiotics exert a beneficial influence on gastrointestinal health and immunological function. Probiotics perform these beneficial actions by changing the kinds of bacteria that live in the gut, altering metabolic function, breaking down chemicals that cause cancer, enhancing the efficacy of immune checkpoint inhibitors, reducing inflammation, and stopping the production of compounds that cause cancer. This current comprehensive review underscores the need for innovative strategies to enhance cancer prevention and treatment, focusing on integrating probiotics into therapeutic approaches to mitigate the adverse effects of conventional cancer therapies, improve patient outcomes, and prevent cancer.

## Summary

- Cancer is a significant global health concern and ranks as the second most common cause of death worldwide.
- An imbalance in gut microorganisms, called dysbiosis, can promote cancer.
- Probiotics have the potential to enhance gut health and restore immunological equilibrium.
- Probiotics exert beneficial effects by altering gut microbiota, modulating metabolism, and reducing inflammation.
- This review highlights the importance of integrating probiotics into cancer prevention and treatment strategies.

## 1 Introduction

Cancer is a significant societal, public health, and economic issue in the 21st century, accounting for over one in six fatalities and ranking as the second most common cause of death globally [1]. Cancer is a lethal malignancy that holds significant clinical implications and is characterized by the unregulated proliferation of an isolated cell, leading to its progression and dissemination throughout the entire organism. Healthy cells undergo a slow transformation into neoplastic cells, progressing through stages of development until they become tumorigenic and ultimately malignant. Certain crucial biological alterations occurring in tumor cells include their ability to produce their own growth signals. They evade apoptosis, multiply indefinitely, create new blood vessels, and spread to other parts of the body [2, 3].

Based on epidemiological research, cancer incidence is expected to increase in the future, which will put pressure on already limited healthcare resources, in addition to being a significant obstacle to extending life expectancy. Hence, it is imperative to develop pragmatic interventions that can efficiently avert and manage many forms of cancer, particularly prevalent ones like colorectal cancer (CRC), prostate cancer, liver cancer, and breast cancer [4]. Moreover, the safety and long‐term sustainability of the standard medications currently used for cancer treatment remain subjects of controversy. Also, these medicines have many adverse effects, and chemotherapy changes hormones in ways that kill healthy cells and make drugs less effective. This makes it even more important to find innovative alternatives to prevent and treat cancer [5, 6].

The investigations confirmed that both genetic and lifestyle factors significantly influence the development of cancer. Approximately 30%–50% of cancer occurrences can be averted by making lifestyle modifications, such as increasing the consumption of healthy foods and vegetables, reducing the intake of processed foods, avoiding smoking and alcohol, and engaging in regular physical activity [7, 8]. Accordingly, the research showed that a crucial and efficient aspect of the cancer prevention and treatment process is the regulation of gut microbiota and the intake of probiotics and their metabolites. The intestinal flora of humans is made up of over 100 trillion symbiotic bacteria, along with much‐outnumbered host cells in the intestine, which are called the gut microbiota [9]. The gut microbiota significantly influences the body's diverse functions, and maintaining its balance is essential to prevent disease and infection caused by pathogens [3, 10]. The gut microbiota is an essential element of human health and has a substantial impact on processes such as nutritional biotransformation, flushing out pathogens, neutralizing toxins, immunological response, and suppressing the development of cancer. Disruption in these relationships can result in gastrointestinal illnesses, neurological diseases, metabolic syndrome, and cancer. Probiotics, obtained from fermented foods or the gut, are utilized in nutritional supplements and pharmaceuticals, and their metabolites provide substantial advantages in preventing and treating cancerous cells [11, 12]. The studies showed that probiotics' anticancer effects come from their ability to control the gut microbiota, change metabolic processes, eliminate factors that result in cancer, change immune responses to lower chronic inflammation, lower the pH of the intestine, and stop the production of compounds that might induce tumors [13, 14]. Probiotics have metabolic effects in the digestive tract, creating compounds like short‐chain fatty acids (SCFAs) and conjugated linoleic acid. Both of these have properties that can help prevent cancer [15]. SCFAs, for example, have been shown to impede the proliferation of cancer cells and induce their demise via apoptosis [16]. Probiotics can also change other things that are known to cause mutations and cancer. They accomplish this by connecting to and dismantling cancer‐inducing molecules in the intestines, reducing the chances that these molecules will cause damage. They can also modulate the immune system, which is essential for recognizing and eradicating cancer cells before their proliferation and dissemination [17, 18]. In this regard, this review explores current and groundbreaking discoveries about the role of probiotics in the prevention, treatment, and mitigation of the adverse effects of anticancer treatments.

## 2 Gut Microbiota's Role in Cancer Development and Immune Response

Cancer can develop as a result of the gradual buildup of genetic mutations and epigenetic alterations in the cellular processes responsible for cell proliferation and death. During this time, genes that promote the growth and survival of cells are turned on, while those that stimulate cell death are turned off. This leads to the development and growth of tumors [19]. Various variables contribute to the occurrence of genetic and epigenetic changes. These include random mutations as well as environmental influences like nutrition, lifestyle, radiation, toxic compounds, pathogens, and alterations in the microbiota of the human body [20]. After that, dying cancer cells and stressed cells send out immunogenic signals that make the immune system fight cancer by activating effector T cell populations. The interactions between commensal bacteria and the host significantly impact the control of the immune system and its response to cancer cells, both directly and indirectly. The composition of the gut microbiota is associated with the onset and progression of cancer and the effectiveness of treatment. These effects are achieved by altering the immune responses to cancers and can be noticed in several types of malignancies, not only in the gut, but also in distant organs [21, 22, 23]. Therefore, the variety and balance of the gut microbiota must remain within a healthy range for homeostasis, or eubiosis, to occur.

## 3 Dysbiosis and Cancer: The Double‐Edged Sword of Gut Microbiota

An imbalance between beneficial bacteria and pathogens can cause dysbiosis. Indeed, the gut microbiota can have both beneficial and detrimental effects. Dysbiosis has the ability to result in a disparity in the interaction between the microbiota and the immune system [24]. This dysregulation has the potential to directly result in chronic inflammation or localized immunosuppression, ultimately leading to the development of neoplasia [25]. The microbiota can either facilitate or hinder tumor formation, depending on the specific conditions. One study that looked at CRC as an important cancer linked to the composition of gut microbiota found that changes in the microbiota of the digestive system have a significant impact on the development of CRC [26]. CRC patients generally exhibit elevated levels of bacteria that produce carcinogenic chemicals, poisons, and bacteria that induce gastrointestinal inflammatory diseases [27]. The microbial composition of fecal samples from patients with CRC also exhibited distinct dissimilarity compared to healthy individuals, specifically characterized by significantly reduced diversity [28]. For instance, some evidence demonstrated that certain *Clostridium* spp. and *Bacteroides* spp. strains have been identified as carcinogens in CRC research. Investigation showed that *Bacteroides fragilis* produces fragilysin, an enterotoxigenic compound, which induces inflammatory mediators that progress the cancer [29]. Also, several studies showed that dysbiosis can increase the likelihood of developing CRC by triggering long‐lasting inflammation and speeding up the production of cancer‐causing chemicals [30, 31]. Sobhani and colleagues compared fecal samples from CRC patients to those of healthy individuals. According to their investigation, the CRC group had significantly larger abundances of *Bacteroides* and *Prevotella* genera [14]. Furthermore, compared to bacteria from the genera *Fusobacterium*, *Eubacterium*, *Prevotella*, *Bacteroides*, and *Proteobacteria*, some *Lactobacillus* species were found in minor quantities in the gut ecosystem. The study also discovered that individuals with CRC had higher concentrations of several *Salmonella* and *Clostridium* species [32]. In addition, the pathogenic strain of *Escherichia coli* can produce a range of toxins, including cytolethal distending toxins and cytotoxic necrotizing factor, as well as other virulence factors that are associated with CRC. Moreover, CRC has also been connected to *Streptococcus gallolyticus* and *Enterococcus faecalis*. The results highlight the need to understand the role of microorganisms in developing CRC and find effective therapies to postpone its onset [33, 34]. The gut microbiome may promote the onset of gastrointestinal cancers through the production of harmful and genotoxic bacterial compounds. These substances bind to receptors on the cell surface and alter intracellular signal transmission pathways [35].

**Figure 1:**
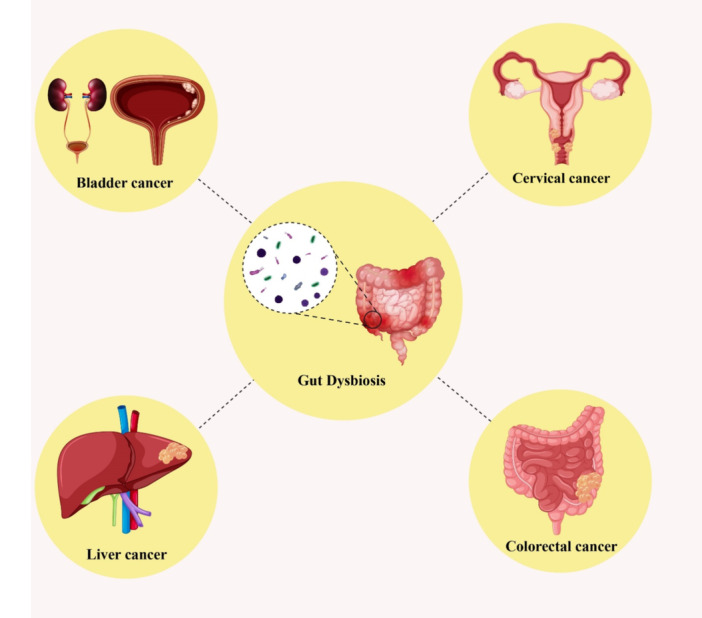
Dysbiosis has been implicated in the pathogenesis of various types of cancer, including breast, colorectal, bladder, and cervical cancers.

The gut microbiota can also influence the other organs through various gut–organ axes. One of these axes is the gut–liver axis. Most of the liver's blood supply is through the portal vein, which carries venous blood from the intestines and spleen. Under normal physiological conditions, the intestinal barrier upholds the gut–liver axis, which effectively restricts the entry of gut microorganisms and their byproducts into the portal vein circulation [36]. Nevertheless, the integrity of the intestinal barrier can be compromised by dysbiosis; consequently, this allows gut bacteria and their byproducts to more readily enter the portal venous circulation and ultimately gain access to the liver. However, certain endotoxins, such as lipopolysaccharide and peptidoglycans, can build up in the liver, leading to hepatic inflammation and the development of liver diseases such as hepatocarcinogenesis (HCC) [37, 38] (Figure 1).

Furthermore, recent evidence has demonstrated that the gut microbiota holds promise as a noninvasive biomarker for cancer detection and management [39]. For example, Zhuang and colleagues discovered a correlation between elevated levels of *Enterococcus* in the gut microbiota and the development of lung cancer. They also identified *Bifidobacterium* and *Enterococcus* as the most promising biomarkers for the initiation of lung cancer. The gut microbiota of lung cancer patients displayed reduced abundance and bacterial diversity compared to healthy persons [40]. More recent primary studies have expanded these findings. For instance, Liu and colleagues carried out a study that showed that patients with lung cancer had reduced levels of *Firmicutes* and *Proteobacteria* while having elevated levels of *Bacteroidetes* and *Fusobacteria* in comparison to healthy individuals [41]. In addition, a case‐control study of gut and lung microbial profiles found decreased microbial richness and distinct compositional shifts in lung cancer patients, including reductions in *Firmicutes* and *Proteobacteria* and relative increases in *Bacteroidetes* and *Fusobacteria*. These findings support the notion that the gut community reflects the disease state and could contribute to diagnostic models [42]. Beyond the gut–lung axis, a study demonstrates that microbiota at other body sites can serve as cancer biomarkers. A 2024 investigation identified a specific *Fusobacterium nucleatum* clade (Fna C2) that is highly enriched in colorectal tumors and exhibits genetic traits enabling tumor colonization, suggesting fecal or tissue detection of this clade could aid CRC identification and stratification [43]. Likewise, a considerable prospective study showed that particular oral bacterial and fungal taxa (including *Porphyromonas gingivalis*, *Eubacterium nodatum*, *Parvimonas micra*, and *Candida* spp.) were associated with later pancreatic cancer risk, indicating that salivary microbiome profiling may provide early‐warning biomarkers for pancreatic malignancy [44]. Collectively, these data demonstrate that distinct microbes at different body sites have biomarker potential for several cancers, but they also highlight substantial heterogeneity between cohorts, the need for standardized sampling and analysis, and the current gap between discovery and validated clinical assays.

## 4 Probiotics: Protection of Gut Health and Immune Balance

According to international definitions, probiotics are composed of living microorganisms that provide health benefits to the individual when consumed in sufficient quantities. The majority of probiotics originate in the digestive tract or from foods that have undergone traditional fermentation. Probiotics have many applications throughout several industries. For instance, when incorporated into nutritional supplements, they are referred to as live microbiological constituents. Also, if they are employed for medicinal purposes, they are known as living biotherapeutic substances [45, 46]. The human gastrointestinal system naturally contains the most prevalent probiotic bacterial groups, including *Lactobacillus* (LAB), *Bifidobacterium* (BFB), *Lactococcus*, and *Enterococcus* [47]. *Bacteroidales*, *Bacillus* species, *Clostridiales*, *Enterococci*, *E. coli*, and *Weissella* species are other possible probiotics [2, 48, 49, 50]. *Bifidobacteria* and lactic acid bacteria have become market leaders after more than a century of rigorous selection. *Lactobacillus* (*paracasei*, *acidophilus*, *gasseri, fermentum*, *casei*, *plantarum*, *johnsonii*, *salivarius*, and *rhamnosus*) and *Bifidobacterium* (*animalis*, *breve*, *adolescentis*, *bifidum*, and *longum*) are the most common species on the market [51].

**Figure 2:**
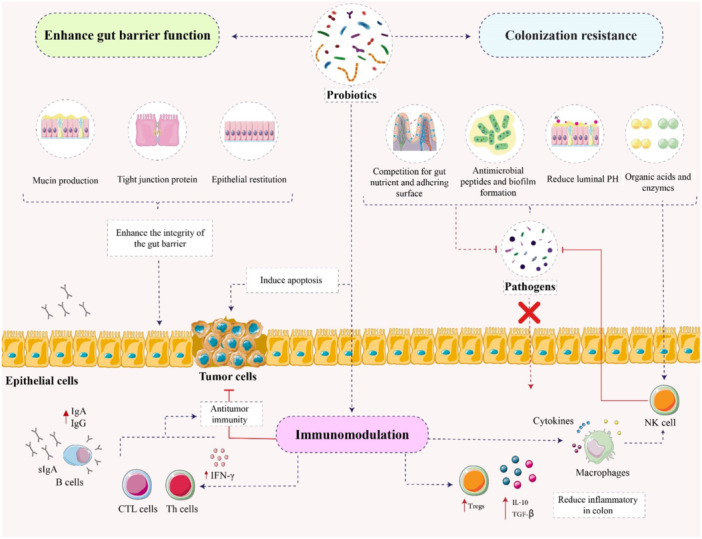
The diagram on display depicts the various mechanisms that may lead to anticancer effect of probiotics through gut tract. Probiotics can exert their antitumor effects through several mechanisms, generally categorized into three processes: enhancing the function of the gut physiological barrier, settling in the digestive tract, inhibiting the colonization of pathogenic microbes, and regulating the immune system through effects on both innate and acquired immunity.

There are accounts of probiotics eliciting both pro‐inflammatory and anti‐inflammatory reactions. While this might appear contradictory initially, it suggests that probiotics play a crucial role in maintaining intestinal balance under various circumstances [52]. Probiotics can modulate cellular, innate, and humoral immunity through different mechanisms [53]. Lactic acid bacteria have specific cellular components that have been shown to stimulate adjuvant effects, such as regulating immune responses through cell‐mediated mechanisms, activating the reticuloendothelial system, and modulating cytokine cascades, interleukins, and tumor necrosis factors [54]. In addition to their capacity to enhance the immune system through various signaling pathways, probiotic bacteria can also regulate and suppress the immune system in specific manners depending on the circumstances, such as infection or inflammation [55]. Furthermore, they can cooperate seamlessly with the gastrointestinal system and release a wide range of enzymes [54]. For example, they can enhance the immune system by producing bile salt hydrolase and exhibit antagonistic effects against infections [56]. Probiotics possess various significant characteristics, such as the ability to adhere to mucosal and epithelial cells, tolerate bile and acid, and resist antibiotics. Probiotics also showed antimutagenic capabilities, and several in vitro investigations have found that probiotics have a beneficial effect on the regulation of cancer cell proliferation and apoptosis [57, 58]. However, probiotic efficacy can vary significantly among individuals, unlike pharmaceuticals. Various physiological impacts of the identical strain on the host have been noted. Multiple variables, including the host's nutrition, age, physical condition, intestinal microbial composition, permission to colonize, and colonization history, influence the extent of the effect [59]. For example, a systematic and comprehensive study found that probiotic supplementation improved progression‐free survival in cancer patients treated with immune checkpoint inhibitors, but the response varied based on individual microbiome compositions [60]. Another study also discussed how personal differences in microbiome composition can significantly influence the effectiveness of administered probiotics, making a one‐size‐fits‐all approach ineffective [61]. Therefore, it is crucial to regulate and maintain the microbiota to ensure the overall health of the host (Figure 2) [62]. These findings underscore the importance of considering individual variability when evaluating probiotic interventions in cancer therapy. Personalized approaches that account for host‐specific factors may enhance the therapeutic potential of probiotics in cancer prevention and treatment.

## 5 Probiotics and Their Immunological Role in Cancer Defense

Probiotics, specifically their SCFAs, are beneficial in the prevention of cancer through many immunological processes. Currently, scientists are studying the immunomodulatory effects and the ability of probiotics to improve the functioning of the immune system [55, 63]. For example, probiotics can stimulate the immune system by enhancing the creation of immunoglobulins, improving the functioning of macrophages and lymphocytes, and increasing the production of pro‐inflammatory cytokines such as interferon‐gamma [64]. Indeed, cytokines can act in two controversial ways. However, the pro‐inflammatory cytokines can enhance the activity of the immune system, but also the chronic inflammation, and the long‐lasting presence of some cytokines, such as IL‐1β, IL‐8, IL‐6, IL‐17, IL‐12, and tumor necrosis factor‐α (TNF‐α), have been associated with the initiation of CRC [65]. Therefore, probiotics can inhibit the proliferation of colon cancer cells by boosting the synthesis of anti‐inflammatory cytokines and reducing the synthesis of pro‐inflammatory cytokines [66]. Also, recent investigations increasingly emphasize that gut microbiota‐derived metabolites (GMMs) exert dual and context‐dependent effects in different cancers, such as CRC, functioning as both tumor promoters and tumor suppressors. On the protective side, SCFAs are frequently reduced in the feces of CRC patients, and their restoration is associated with beneficial immunometabolic outcomes [67]. SCFAs are considered to be one of the significant bacterial metabolites. These fatty acids, including propionic, acetic, butyric, caproic, and valeric acids, are produced by bacteria that inhabit the large intestine [68]. SCFAs provide energy to colon cells and help in maintaining the appropriate balance between cell division, proliferation, and apoptosis. They promote the creation of an acidic environment, which can inhibit the growth of cancer cells and inhibit the excessive production of secondary bile acids [69]. Patients with CRC often have decreased levels of butyrate in their feces compared to healthy individuals. An investigation showed that a drop of 1 µg/L in butyrate concentration in feces is associated with an 84.2% increase in the risk of colon cancer, while a fall of a similar magnitude in acetic acid concentration is connected to a 71.3% increase in the possibility of developing adenoma [32]. Although SCFAs are produced by the gut microbiota, the amount generated might not be enough to stop CRC from developing. As a result, consuming probiotics can potentially increase the production of SCFAs regularly.

In this regard, a study showed that SCFAs act through histone deacetylase (HDAC) inhibition and reinforcement of intestinal barrier integrity [70]. Similarly, secondary bile acids such as ursodeoxycholic acid (UDCA) have been shown to exert antitumor effects; for example, UDCA reduces immunosuppression by inducing CHIP‐mediated degradation of TGF‐β, thereby restraining regulatory T cell differentiation and enhancing responsiveness to anti‐PD‐1 therapy [71]. In addition, tryptophan metabolites such as kynurenic acid and indole derivatives can contribute to immune regulation and suppression of inflammatory signaling, thereby attenuating CRC progression [72]. A 2024 study using *Lactobacillus reuteri* delivered via a microgel showed increased levels of butyrate, activation of GPR109A on colonic epithelial cells, and induced apoptosis in CRC models [73]. In addition, in metastatic CRC patients, combining probiotics (*Bifidobacterium* triplet living bacteria tablets) with immune checkpoint inhibitors and chemotherapy significantly improved measures of intestinal immunity and clinical prognosis, demonstrating the translational potential of probiotic‐immune synergy [74]. Metagenomic and metabolomic analyses in CRC patients have also shown that a decline in butyrate‐producing bacteria and a reduction in acetate correlate with worse outcomes; experimental loss of the SCFA receptor FFAR2 (GPR43) in mouse models led to increased tumor growth, compromised barrier function, and immune cell exhaustion [75].

Studies also showed that probiotics can reduce inflammation in the intestines by decreasing the expression of TLR, producing metabolites that prevent TNF‐α from reaching blood mononuclear cells and inhibiting NF‐κB activation in enterocytes [76]. In this regard, a study conducted in 2017 demonstrated that administration of *Bifidobacterium infantis* resulted in a drop in the levels of IL‐6, IL‐1β, and TNF‐α, which had been elevated due to chemotherapy [77]. The results can support the immunomodulatory capabilities of probiotics and their efficacy in reducing cancer symptoms and mitigating chemotherapy adverse effects [78]. In addition, multiple studies have demonstrated that the cell wall constituents of lactic acid bacteria can enhance the performance of macrophages by increasing the production of lysosomal enzymes and free oxygen radicals [79]. They cause macrophages to release cytokines, which in turn cause host natural killer cells and cytotoxic T cells to become active in the immune system [80].

Conversely, several GMMs exhibit tumor‐promoting functions. Among these, secondary bile acid derivatives like deoxycholic acid (DCA) and conjugated bile acids are strongly implicated in inflammation and DNA damage, while hydrogen sulfide (H_2_S), generated by gut microbes such as *F. nucleatum*, disrupts autophagy and promotes CRC progression [81]. Likewise, elevated trimethylamine *N*‐oxide and certain tryptophan catabolites contribute to pro‐tumorigenic immunosuppression and foster a microenvironment conducive to cancer cell proliferation. Moreover, bacterial genotoxins such as colibactin and adhesins directly damage host DNA and further support oncogenesis [82, 83]. Collectively, these findings demonstrate that while certain GMMs reinforce antitumor immunity and epithelial homeostasis, others exacerbate inflammation, genomic instability, and immune evasion, highlighting the necessity for precise, context‐driven therapeutic strategies.

## 6 Revolutionizing Cancer Therapy and Prevention

### 6.1 Mechanism of Probiotic Action

Currently, the field of cancer treatment encounters obstacles and adverse effects, such as harm to healthy tissues, susceptibility to infections, and enduring consequences such as organ failure. Conventional methods such as surgery, chemotherapy, and radiation can have adverse effects, including nausea, vomiting, hair loss, and fatigue. Cancer cells exhibit adaptability and resistance to therapies, rendering them ineffectual. Additionally, individuals may experience psychological consequences such as anxiety, depression, and a heightened dread of the condition returning. Researchers are investigating treatments that have improved specificity and heightened immune system potency to reduce the side effects of treatment and boost overall quality of life. In this regard, research has shown that probiotics can serve as a supplementary approach to cancer treatment and help mitigate the adverse effects of traditional therapies (Table 2) [84, 85].

#### 6.1.1 Strengthening Gut Integrity to Ward Off Cancer

As previously stated, probiotics have the potential to modify the composition of the gut microbiota, thereby shifting the equilibrium of bacteria in the gut toward a more favorable condition, resulting in a decreased possibility of developing cancer. Probiotics can enhance the integrity of the intestinal barrier, thus inhibiting the entry of detrimental compounds into the bloodstream and mitigating the potential for cancerous injury. They can also help by posing competition for intestinal mucosa and nutrient adherence with pathogenic microbes [86]. Probiotic strains can generate biofilms, structural matrices made of extracellular polymers produced by microbes that adhere to a surface and aid in adhesion [87]. Structural variety, genetic diversity, interaction complexity, and the presence of extracellular materials such as polysaccharides, proteins, phospholipids, and nucleic acids are characteristics of biofilms. These substances arise due to environmental adaptation, and polysaccharides, which fill the spaces among microorganisms in biofilm, are crucial for adhesion [88, 89]. The clinical trial carried out by Ohara and colleagues for 12 weeks on patients with CRC revealed a direct correlation between the abundance of *Lactobacillus gasseri* OLL2716 (LG21) and the production of isobutyric acid. Consequently, the rise in isobutyric acid resulted in heightened NK cell activity and a reduction in the presence of *Clostridium perfringens* in these patients [90]. This implies that by enhancing the gut ecology, consuming probiotics may help prevent CRC. In CRC patients who require surgery, Kotzampassi and colleagues discovered that probiotics (*Bifidobacterium lactis, Lactobacillus acidophilus plantarum*, and *Saccharomyces boulardii*) notably decrease the risk of postsurgical complications, including infections, anastomotic leakage, and mechanical ventilation [52]. In addition, a study was conducted via an animal model of CRC induced by subcutaneous injection of dimethyl hydrazine (DMH). The study compared the effects of chemotherapy (Oxaliplatin) alone with the combination of Oxaliplatin and the probiotic *B. infantis*. The study findings demonstrated that the consumption of probiotics led to a significant rise in body weight and height of intestinal villi [77]. Considering everything, using probiotics to enhance gut microbiota can benefit the host, especially in reducing the growth of gastrointestinal malignancies.

#### 6.1.2 Controlling Cancer Cell Death

Studies have demonstrated that probiotic bacteria control cell death via intrinsic and extrinsic pathways, which may be essential in cancer prevention [65]. Goldin and Gorbach were pioneers in proposing a connection between a diet enriched with *Lactobacillus* and a 37% reduction in the incidence of CRC compared to the control group [91]. Numerous in vitro studies have highlighted the beneficial effects of probiotics in regulating the growth and programmed cell death of various cancer cells, such as those found in the stomach, colon, and myeloid leukemia. Most of these laboratory experiments have concentrated on studying human colon cancer cells [53, 92]. Numerous researchers have seen a noteworthy impact in suppressing cell growth and/or stimulating programmed cell death in both mouse colon carcinoma (HGC‐27) and human colon cancer cell lines (Caco‐2, HT‐29, DLD‐1), and also reducing the IL–8 levels [93, 94, 95, 96, 97, 98]. Additionally, research reports highlight the efficacy of various probiotic microorganisms, including *Bacillus* species like *polyfermenticus* and *subtilis*, *Bifidobacterium* species such as *lactis* and *adolescentis, Lactobacillus* species like *casei*, *acidophilus*, *fermentum*, *delbrueckii*, and *plantarum*, as well as *Lactococcus lactis*, *Propionibacterium acidopropionici*, and *Streptococcus thermophilus*. These probiotics have demonstrated efficacy in reducing the proliferation and/or inducing apoptosis in human colonic cancer cells such as DLD‐1, HCT116, SW1116, SW480, Caco‐2, LoVo, and HT‐29 [99].

Furthermore, *L. acidophilus* CL1285 and *Lactobacillus casei* LBC80R, particularly in combination with 5‐fluorouracil (5‐FU), have been found to induce apoptosis in human colorectal cells (LS513) [100, 101]. In another study, Chen and colleagues found that administering *L. acidophilus* orally in mice decreased the intensity of CRC and enhanced apoptosis through downregulating NF‐kB‐dependent gene products, which control cell proliferation (cyclin D1, Cox‐2) and survival (Bcl‐2, Bcl‐xL), leading to apoptosis [102]. In addition, another investigation showed that *L. reuteri* can slow down cancer cell growth by suppressing TNF‐induced NF‐kB activation and may play a role in the extrinsic pathway of apoptosis [102]. In this regard, Cousin and colleagues suggested another mechanism for probiotic‐induced apoptosis by showing that *Propionibacterium freudenreichii* can trigger the formation of apoptotic bodies, chromatin condensation, DNA fragmentation, activation of caspases, disruption of mitochondrial transmembrane potential, buildup of reactive oxygen species (ROS), and halting of the cell cycle in HGT‐1 gastric cancer cell lines [103]. Furthermore, Baldwin and colleagues conducted research on the effectiveness of *L. acidophilus* and *L. casei* in inhibiting the spread of LS513 gastric cancer cells. It was shown that the two probiotic bacteria exhibited a higher capacity to trigger cell death in the presence of conventional chemotherapy drugs like 5‐FU. This could be attributed to the accelerated activation of the caspase‐3 protein and a decrease in the levels of the p21 protein [100]. In addition to gastrointestinal and especially CRCs, probiotics can act as a treatment method for other cancers by increasing the apoptosis of cancer cells. For instance, the HPV E6 and E7 proteins play a critical role in the development of cervical cancer by inactivating the p53 and pRb tumor suppressor genes, which inhibit cell death and impair the immune system's ability to detect cancer cells [104, 105]. The research also conducted on the SiHa cell cervical cancer lineage showed that *B. adolescentis* SPM1005‐A can suppress the expression of HPV E6 and E7 oncogenes, indicating that it may be used to prevent HPV‐associated cervical cancer [106].

#### 6.1.3 Optimizing the Therapeutic Effect of PD‐1 Checkpoint Inhibitors

Research has emphasized the correlation between immunotherapy utilizing immune checkpoint inhibitors, such as PD‐1/PD‐L1, and the microbiota [13]. Multiple studies have shown a significant correlation between the amount, composition, and kind of gut bacteria in individuals with cancer and the efficacy and survival of those individuals on PD‐1 inhibitor treatment. One potential explanation for the mechanism is that the interaction between various types of microorganisms in the body boosts the innate immune system's capacity to defend against cancer [107, 108].

Routy and colleagues examined the impact of PD‐1 immunotherapy on individuals diagnosed with bladder, kidney, and lung cancer. A study revealed that the effectiveness of immunotherapy was considerably diminished when patients had been administered broad‐spectrum antibiotics before and following the commencement of treatment (2 months before and 1 month after). This was ascribed to disturbances in the body's microbiota, specifically the gut microbiota. In contrast, individuals who did not receive broad‐spectrum antibiotic treatment had significantly increased rates of progression‐free survival and overall survival. PD‐1 blocking treatment elicited a positive response in certain individuals, likely attributed to the presence of the gut‐dwelling bacterium *Akkermansia muciniphila* [109]. Gopalakrishnan and colleagues discovered in a different clinical investigation that the distinction and composition of trillions of harmful and beneficial bacteria in the gastrointestinal tract influenced how melanoma patients responded to anti‐PD‐1 treatment. Through the study of stool samples from patients receiving PD‐1 checkpoint inhibitor medication, it was discovered that these patients had more diverse gut flora and higher levels of *Clostridium* order in comparison to those who did not respond to the treatment. Individuals with melanoma who have a high concentration of *Bacteroides* bacteria in their intestines tend to have significantly lower bacterial diversity compared to those whose melanoma has responded to therapy [108]. In these cases, probiotics have the potential to efficiently control the population of bacteria, making them a promising approach for the prevention or treatment of cancer.

#### 6.1.4 Prevention of Cancer by Fighting Against Carcinogenic Compounds

Probiotics not only have a substantial influence on cancer patients' treatment but also can possess the capacity to prevent cancer development through many mechanisms. In a dysbiosis condition, certain populations such as *Bacteroides*, *Clostridium*, *E. coli*, and *Eubacterium* exhibit heightened enzyme activity involved in the production of cancer‐causing substances [110]. Research demonstrated that these gut microbiota can produce several enzymes like nitrate reductase, β‐glucosidase, azoreductase, and β‐glucuronidase to metabolize food toxins and bile salts produced within the body [111]. These enzymes have the ability to generate ammonia, cresols, aglycones, N‐nitroso compounds, and phenols. Additionally, they can convert heterocyclic aromatic amines, primary bile acids, and polycyclic aromatic hydrocarbons into potent carcinogens [65]. These metabolites have the ability to damage DNA and cells, which can lead to abnormal cell proliferation and activation of pathways that prevent cell death. Ultimately, this can contribute to the development of CRC [30]. A clinical trial conducted by Hatakka and colleagues demonstrated that administering *Lactobacillus rhamnosus* LC705 and *P. freudenreichii* ssp. *shermanii JS* to healthy men for 4 weeks resulted in a reduction in urease and β‐glucosidase activity, as well as an increase in the population of *Lactobacillus* and *Propionibacterium* bacteria [112]. Another study has shown that *L. acidophilus* had a beneficial effect on the functioning of azoreductase, nitroreductase, and β‐glucuronidase in a group of 21 healthy adults. The fecal enzymes can transform procarcinogens into proximal carcinogens. Following 10 days of probiotic consumption, the *lactobacillus* strains effectively decreased the specific activity of the three enzymes in all patients [113].

Moreover, probiotics can impact factors that are carcinogenic and mutagenic by regulating the function of specific enzymes responsible for cellular detoxification. They can effectively suppress the release of detrimental compounds, such as carcinogens and free radicals [114]. The antioxidant enzyme glutathione S‐transferase (GST) neutralizes dangerous substances like ROS and xenobiotics that may trigger cancer and also has detoxifying qualities. GST shields DNA from oxidative stress, which can cause mutations and, eventually, cancer [115]. Through the metabolic process of butyrate, which can change the status of histone acetylation and boost GST expression, probiotics may enhance the activity of GST [116]. In the colon, β‐glucosidase hydrolyzes plant glycosides like cycasin to produce carcinogens called aglycones [117]. Clinical research indicates that probiotics exhibit comparable inhibitory effects on these cancer‐causing enzymes. Research, for example, showed that *Bifidobacterium* species decreased the intestinal β‐glucuronidase activity [118]. In a CRC animal model, Chandel, along with colleagues, discovered that the use of *L. acidophilus, L. rhamnosus GG*, or a combination with celecoxib decreased NF‐kB, β‐catenin, COX‐2, and K‐ras carcinogenic biomarkers [28]. Hence, the consumption of beneficial probiotic microorganisms might diminish the presence of detrimental bacteria in the gut microbiota, thereby decreasing the concentration of cancer‐causing chemicals in the gut [119].

Furthermore, such studies demonstrate that several probiotics have the ability to adhere to and metabolize carcinogenic compounds present in the intestines through the production of specific molecules. According to research, some probiotic bacterial strains, such as *L. acidophilus*, *Bifidobacterium longum*, and *Streptococcus salivarius*, can attach to carcinogenic substances by exchanging ions with peptidoglycan found in some probiotic microorganisms' cell walls. This binding may result in the release of mutagens and heterocyclic amines, including 3‐amino‐1‐methyl‐5H‐pyrido [4,3‐b] indole acetate (TrpP2), 5‐phenyl‐2‐amino‐l‐methylimidazo [4,5‐f] pyridine (PhMIP), 2‐amino‐3,4‐dimethylimidazo [4,5‐f] quinoline (MeIQ), and 2‐amino‐3‐methyl‐3H‐imidazo [4,5‐f] quinoline (MHIQ) [120, 121]. In their investigation of the impact of gut microbiota on dimethyl‐nitrosamine, Rowland and Grasso discovered that *Lactobacillus* bacteria exhibited the highest degree of efficacy in degrading the chemical [122]. Dimethylamine, the amine's precursor, and other volatile metabolites were produced from it. Additionally, *L. casei* has shown a significant ability to neutralize carcinogenic heterocyclic amines, as demonstrated by Morotomi and Mutai. According to their research, this capability was exclusively exhibited by live bacteria, suggesting that these organisms catalyze or generate metabolites that lead to the detoxification of amines [123]. Furthermore, research suggests that probiotics could detoxify mycotoxins, which have the potential to cause malignancies. El‐Nezami and colleagues, for example, have shown that a 5‐week probiotic supplementation can lower the urine excretion of aflatoxin B(1)‐N(7)‐guanine, a marker for hepatocyte tumorigenesis [124, 125].

### 6.2 Preserving Gut Health via Antimicrobial Action

Research indicates that a key characteristic of lactic acid probiotics is their ability to produce metabolites that are essential for regulating the balance of gut microbiota and inhibiting the growth of pathogenic bacteria. The most important compounds include bacteriocins, hydrogen peroxide, acetic and lactic acid, and organic acids. These molecules hinder the growth, activation, and multiplication of pathogens by lowering the pH of the environment [126, 127]. Also, due to its chemical properties, lactic acid can penetrate the cell membrane and, upon entering the cell, it interferes with the proton gradient in the membrane. This ultimately leads to acidification of the cytoplasm in the pathogenic bacterium cell, resulting in the cessation of microbial growth [128]. Furthermore, small amounts of other acids, such as propionic, formic, and benzoic, have the potential to produce a synergistic effect [129]. Hydrogen peroxide is able to successfully restrict or eradicate bacteria that have low levels of enzymes that break down H_2_O_2_, due to its strong oxidizing properties. This prevents the oxidation of proteins within the cells. However, it can also eradicate strict anaerobes and beneficial gut bacteria [130, 131]. Bacteriocins, synthesized by probiotics, have the ability to destroy or inhibit the growth of sensitive microorganisms by damaging their cell membranes. Bacteriocins have the power to interfere with the synthesis of DNA, RNA, and proteins. They can also alter the transmembrane potential, leading to the leakage of ions and amino acids from the cytoplasm of infected cells [132, 133]. Bifidocin B, nisin, and lactacin B are three specific bacteriocins. Despite their limited antibacterial activity, bacteriocins in the gastrointestinal system may have a role in quorum sensing or host immunological control [134, 135, 136].

### 6.3 The Role of Probiotics in Reducing Cancer Treatment Complications

Gastrointestinal discomfort is a prevalent adverse effect of cancer treatment. Radiotherapy and chemotherapy target intestinal cells directly, leading to stress‐induced damage to the intestinal mucosal barrier. Increasing intestinal mucosal permeability allows the gut microbiota and endotoxins to infiltrate other tissues and organs, leading to systemic inflammation and potentially causing multiple organ failure [137, 138]. Multiple studies have demonstrated that delivering probiotics to patients before abdominal surgery can effectively reduce the likelihood of infection and other adverse consequences, which are significant factors contributing to patients' morbidity. In a 2019 prospective randomized controlled trial, researchers explored the efficacy of probiotics on the health condition of cancer patients following surgery for CRC. The findings of this study demonstrated a disparity in the advantages of utilizing probiotics during hospitalization following surgery and within the initial 6 months. Specifically, the group of patients who did not receive probiotic treatment experienced various complications, including surgical site infections, loosening of anastomosis, intra‐abdominal abscess, and ileus [18]. Moreover, some probiotic microorganisms are useful for controlling a variety of intestinal problems, such as fever, postoperative inflammatory diseases, viral diarrhea, and diarrhea caused by antibiotics or chemotherapy/radiotherapy [57, 125, 139, 140, 141, 142, 143, 144]. In 2013, research conducted by Liu and colleagues demonstrated that administering *Bifidobacterium longum*‐88, *Lactobacillus acidophilus*‐11, and *Lactobacillus plantarum* CGMCC to 150 CRC patients for 6 days before surgery and 10 days after surgery resulted in several positive outcomes. These included decreased postoperative fever duration, decreased incidence of postoperative infectious complications, shortened antibiotic therapy duration, reduction in serum zonulin concentration, and inhibition of the p38 mitogen‐activated protein kinase signaling pathway [145]. The study undertaken by Zaharuddin and colleagues explored the impact of consuming probiotics containing six live microorganisms, including *Lactobacillus* and *Bifidobacteria* strains, for 6 months on the clinical outcomes and inflammatory cytokines in patients diagnosed with CRC. The results indicated that although both groups experienced chemotherapy‐induced diarrhea, there was a significant decrease in the levels of pro‐inflammatory cytokines (TNF‐α, IL‐6, IL‐10, IL‐12, IL‐17A, IL‐17C, and IL‐22) in CRC patients who received probiotics compared to their levels before surgery. Furthermore, there were no instances of postoperative infection and no need for antibiotics in either group [146]. Furthermore, previous studies have demonstrated that neoadjuvant chemotherapy (NACT) can result in an increased occurrence of complications following surgery. In a 2022 investigation examining the impact of probiotics on postoperative infections and other short‐term outcomes in gastric cancer patients receiving NACT. In this study, the probiotic group showed a significant reduction in postoperative infections, faster time to first flatus and bowel movement, and lower inflammatory indexes compared to the control group [147]. While the results are promising, caution is necessary when interpreting them because most of the tumors were induced by specific chemical agents, which significantly deviates from the normal process of carcinogenesis [148] (Table 1).

**Table 1:** Managing chemotherapy side effects with probiotics.

Probiotic	Chemotherapy side effect	Cancer type	Number of patients	Study conclusion	Reference
*Lactobacillus*	Diarrhea	Various	100	*Lactobacillus* supplementation reduced the severity and duration of diarrhea in cancer patients undergoing chemotherapy	Österlund et al. [142]
*Bifidobacterium*	Gastrointestinal discomfort	Various	80	*Bifidobacterium* supplementation alleviated gastrointestinal discomfort in cancer patients receiving chemotherapy	Rodriguez‐Arrastia et al. [149]
*Saccharomyces*	Diarrhea, GI upset	Various	60	*Saccharomyces* helped in reducing diarrhea and gastrointestinal upset in patients undergoing chemotherapy	López‐Gómez et al. [150]
*Streptococcus*	Oral mucositis	Head and neck	50	*Streptococcus* supplementation decreased the severity of oral mucositis in head and neck cancer patients receiving chemotherapy	Peng et al. [151]
*Enterococcus*	Diarrhea	Colorectal	40	*Enterococcus* supplementation reduced the incidence and severity of diarrhea in colorectal cancer patients undergoing chemotherapy	Feng et al. [152]
*Escherichia*	Infections	Various	30	*Escherichia* supplementation helped in preventing infections in cancer patients undergoing chemotherapy	Mego et al. [153]
*Bacillus*	Diarrhea, gastrointestinal discomfort	Various	15	*Bacillus* supplementation mitigated diarrhea and gastrointestinal discomfort in cancer patients undergoing chemotherapy	Wei et al. [154]
*Pediococcus*	Diarrhea	Colorectal	10	*Pediococcus* supplementation decreased the incidence of diarrhea in colorectal cancer patients receiving chemotherapy	Sivamaruthi et al. [155]
*Clostridium*	*Clostridium difficile* infection	Various	5	*Clostridium* supplementation reduced the risk of *Clostridium difficile* infection in cancer patients undergoing chemotherapy	Francisco et al. [156]

**Table 2:** Synopsis of probiotics' role in cancer therapy and prevention.

Aspect	Mechanism of action	Probiotic strains	Experimental context
Strengthening gut integrity	Enhances microbiota composition, intestinal barrier, inhibits pathogens, reduces carcinogenic metabolites	*L. gasseri OLL2716, B. lactis, S. boulardii*	CRC prevention and postsurgical recovery
Clinical trial insights	Improved NK cell activity; reduced complications in CRC surgery	*L. acidophilus, L. plantarum, B. infantis*	CRC surgical patients and CRC animal models
Biofilm contribution	Structural matrices aid adhesion, competition for nutrients, pathogen inhibition	General probiotic biofilm‐producing strains	Gut health and intestinal integrity improvement
Chemotherapy enhancement	Combines with Oxaliplatin to increase body weight and villi height	*B. infantis*	Animal model (DMH‐induced CRC)
Cancer cell death	Induces apoptosis through NF‐κB suppression, caspase activation, ROS buildup, and cell cycle arrest	*L. acidophilus, P. freudenreichii, L. casei*	Various cancer cell lines (colon, gastric, cervical)
Optimizing immunotherapy	Enhances immunotherapy efficacy by modulating gut microbiota composition and diversity Broad‐spectrum antibiotics disrupt microbiota and reduce therapy outcomes	*A. muciniphila, Clostridium* spp., *Bacteroides*	Melanoma, bladder, kidney, and lung cancer patients
Carcinogenic compound defense	Reduces enzyme activities, lowering carcinogen formation	*L. rhamnosus, P. freudenreichii, L. acidophilus*	Clinical trials and healthy adult studies
Detoxification and antioxidant roles	Neutralizes ROS, enhances GST expression, prevents oxidative stress‐induced mutations	General probiotic strains	Detoxification and antioxidant protection in the gut

## 7 Suggestions and Future Investigations

A very large human database is needed to identify the best strains for the prevention and treatment of various cancer types, and a comprehensive analysis of the relationships between strains and clinical outcomes is also required. The subsequent challenge involves determining the optimal approach for leveraging probiotics and their byproducts to modulate patient microbiota once a beneficial flora for cancer prevention and treatment has been identified. In addition, because of the response of gut flora to modifications in the pathophysiological milieu, it may be utilized as a novel cancer biomarker. Finding particular strains or combinations of strains that can improve anticancer treatment and lessen the negative effects of cancer treatment is the ultimate goal. Thus, in the future, the control of specific human flora is probably going to be a new area of precision and personalized treatment for diseases like cancer.

## 8 Conclusion

In conclusion, the burgeoning field of probiotics presents promising avenues for both cancer treatment and prevention. Through various mechanisms such as modulation of the gut microbiota, regulation of inflammation, and enhancement of the immune system, probiotics demonstrate potential in combating cancer. Moreover, they serve as valuable adjuvants in traditional cancer therapies, potentially augmenting treatment efficacy. Probiotics also exhibit preventive properties, with evidence suggesting their ability to inhibit tumor growth and prevent cancer initiation. Notably, probiotic metabolites have been implicated in epigenetic mechanisms that contribute to colon cancer prevention. As ongoing research expands our understanding of the intricate relationship between probiotics and cancer, the potential for innovative therapeutic approaches continues to grow. Harnessing the power of probiotics may offer new avenues for personalized cancer care, ushering in a future where these beneficial microorganisms play a vital role in our fight against cancer.

## Author Contributions

Mostafa Saebi, Amir Hossein Barjasteh, Setayesh Ramazanpour, and Negar Ebadpour participated in data collection, performing the project, and manuscript writing. Ramiar Kamal Kheder and Afsane Fadaee participated as grammatical editors. Seyed‐Alireza Esmaeili and Mahmoud Mahmoudi designed and drafted the article. All authors have fully read and approved the final manuscript. This manuscript has been read and approved by all the authors, and all authors listed on the manuscript have agreed to its submission. Additionally, all of the authors have approved the contents of this paper and have agreed to the journal's submission policies.

## Conflicts of Interest

The authors declare no conflicts of interest.
